# Associations between thyroid dysfunction and developmental status in children with excessive iodine status

**DOI:** 10.1371/journal.pone.0187241

**Published:** 2017-11-22

**Authors:** Inger Aakre, Tor A. Strand, Khalil Moubarek, Ingrid Barikmo, Sigrun Henjum

**Affiliations:** 1 Department of Nursing and Health Promotion, Faculty of Health Sciences, Oslo and Akershus University College, Oslo, Norway; 2 Department of Global Public Health and Primary Care, Faculty of Medicine, University of Bergen, Bergen, Norway; 3 Research Department, Innlandet Hospital Trust, Lillehammer, Norway; 4 Saharawi Ministry of Public Health, Rabouni, Algeria; Hokkaido Daigaku, JAPAN

## Abstract

**Background and objective:**

Adequate iodine status and normal thyroid hormone synthesis are important for optimal child development. In this study, we explored whether young children’s developmental status is associated with thyroid dysfunction in an area of chronic excessive iodine exposure.

**Methods:**

We included 298 children between 18 and 48 months of age residing in Algerian refugee camps. Early child development was measured using the Ages and Stages Questionnaires, third edition (ASQ-3), consisting of five domains: Communication, Gross Motor, Fine Motor, Problem Solving and Personal-Social. Due to poor discriminatory ability in the Gross Motor domain, the total ASQ-3 scores were calculated both including and excluding this domain. Urinary iodine concentration (UIC), thyroid hormones (TSH, FT3 and FT4), thyroid antibodies and serum thyroglobulin (Tg) were measured.

**Results:**

The median UIC was 451.6 μg/L, and approximately 72% of the children had a UIC above 300 μg/L. Furthermore, 14% had thyroid disturbances, of whom 10% had TSH outside the reference range. Children with thyroid disturbances and TSH outside the reference ranges had lower odds of being among the 66% highest total ASQ scores, with adjusted odds ratios (95% CI) of 0.46 (0.23, 0.93) and 0.42 (0.19, 0.94), respectively.

**Conclusion:**

We found an association between thyroid dysfunction and poorer developmental status among children with excessive iodine intake. The high iodine intake may have caused the thyroid dysfunction and hence the delayed developmental status; however, other influential factors cannot be excluded. Optimal child development is important for a sustainable future. With iodine excess being an increasing problem globally, this subject should be further explored.

## Introduction

Iodine in an essential trace element that is required for the synthesis of the thyroid hormones, thyroxin and triiodothyronine [1]. Thyroid hormones are important for the synthesis of growth hormones, cell differentiation, and the development of the central nervous system. Therefore, iodine is crucial for the growth and development of children [2, 3]. Particularly during fetal life, brain development is dependent on sufficient thyroid hormones for neurogenesis, neural migration, the growth of axons and dendrites, the formation of synapses and myelination [4]. Both iodine deficiency and iodine excess are known to interfere with thyroid hormone synthesis [5–8]. While severe iodine deficiency has been eliminated, the number of countries with iodine excess has increased over the past decade [9–12]. Fetuses and newborn children are particularly vulnerable to iodine excess, since their immature thyroid gland is unable to escape from the Wolff-Chaikoff effect [13–15], which may lead to long-term thyroid dysfunction [6].

Iodine deficiency is known to increase the risk of impaired mental function and delayed physical development in children, due to disturbances in thyroid hormone production [1].

The relationship between severe and moderate iodine deficiency and poor developmental outcomes has been established [3, 5, 16]. In a Lancet series about Child Development, iodine deficiency was listed as one of four main factors that prevent children from achieving their developmental potential [17]. Although thyroid hormone status may be affected similarly by iodine deficiency and excess, there is a gap in the scientific literature regarding the impact of iodine excess on child development. A Chinese study found an association between high iodine intake from drinking water and lower IQ-scores among children [18]. In another study from China, increased iodine intake among schoolchildren was associated with an increased risk of subclinical hypothyroidism but not with any obvious effects on mental development [19]. We have previously found a high prevalence of thyroid dysfunction among children living under chronic iodine excess [20]. The high iodine intake in this population was found to probably originate from high iodine in drinking water and animal milk [21, 22]. In this paper, we explore whether young children’s developmental status measured by the Ages and Stages Questionnaires, third edition, is associated with thyroid hormone status in an area of chronic excessive iodine exposure. This is one of the first papers to explore the relationship between child development and thyroid function in an area of excess iodine.

## Methods

### Subjects

**Fig 1:**
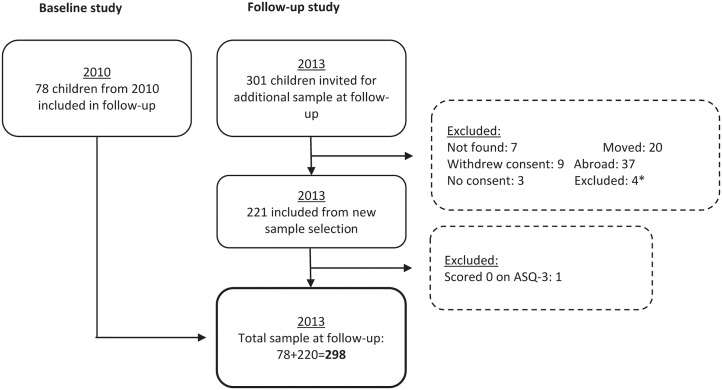
Flowchart for selection of Saharawi children from 2010 baseline study and 2013 follow-up study. *low age, high age, deaf, and mute.

This cross-sectional study was performed in 2013 in the Saharawi refugee camps, which are located in the south-west of the Algerian desert. The sample size was estimated based on an effect size of 20 in the ASQ score, which was found to be clinically relevant, and a standard deviation of ± 50. This would require a sample size of 132 children in each group and a total number of 264 children, using a power of 90% and a significance level of 5%. Approximately 10% was added to account for the possibility that there may be some missing data and that the goiter-to-non-goiter ratio might deviate from 1:1 [23]; thus, we arrived at a final sample size of 298 children. Among the 298 children, 78 were included from a baseline study in 2010 and 220 children were randomly selected in 2013 from a census of all children born between 2010 and 2011 in the refugee camps. All children born in this time-span were included in the selection procedure. None of the selected children had known thyroid disease, or was being treated with thyroid hormones. The selection procedure for the 2010 study has been previously described [20], and the selection procedure for the 2013 follow-up is presented in Fig 1.

### ASQ-3

The Ages and Stages Questionnaires, third edition (ASQ-3), is a comprehensive standardized developmental screening tool for children from 1–66 months of age. The ASQ-3 are developed to be a self-administered tool completed by caregivers but may also be administered by trained personnel, as described in the “home visit procedure” in the ASQ-3 user manual [24]. The instrument consists of age-specific questionnaires, each containing 30 items distributed over five subscales: Communication, Gross Motor, Fine Motor, Problem Solving and Personal-Social. Scores in each subscale range from 0–60, and the total score range from 0–300.

### Procedure

Ten of the age-specific questionnaires from the ASQ-3 were used in this study, which included the ages of 18, 20, 22, 24, 27, 30, 33, 36, 42 and 48 months. Before the fieldwork, an expert group consisting of a clinical psychologist specialized in child psychology, a Saharawi woman, and the field supervisor, reviewed the questionnaires from the ASQ-3 used in this study. All items used were carefully reviewed regarding the feasibility for the study population. Some adjustments were made to several items to better fit the cultural environment. All adjustments were also discussed with the research team and the field group, which consisted of fieldworkers, a trained ASQ-3 administrator, and the field supervisor. The ASQ-3 were formally translated from English into the local language of Hassaniya. The translation process followed the recommended procedure for translation of instruments by the WHO [25]. The field workers were trained by an experienced ASQ administrator and the field supervisor for 11 days prior to conducting the ASQ-3. The training consisted of achieving an understanding of the instrument and the different items, the terms of scoring, and practical training using the ASQ-3 on a test sample. Standardization exercises were conducted to ensure that the fieldworkers had an appropriate inter-observer agreement. The questionnaires were pilot tested on a small sample population.

### Administration of ASQ-3

The ASQ-3 was assessed at the home of each participant, using the “home visit procedure” [24]. The examiners obtained the items by interview with caregivers and observation during the sessions. All necessary equipment was standardized, and brought by the examiners, to ensure that the same equipment was used for each session. Before the assessment, each child was given 20 minutes to play with the material and feel safe with the examiner, while the mother was interviewed for background information. The mothers were interviewed using a pre-coded questionnaire regarding background information and home environment.

### Interobserver reliability

During the assessments, we measured interobserver reliability in 52 (17.4%) of the child participants in the study. These measurements were obtained by scoring the same session by two examiners. The examiners rotated among themselves, and the children were randomly selected for double scoring throughout the study. All age groups were represented, except the age group of 48 months, which consisted of only 4 children. Intraclass correlations (ICC) between the different examiners were consistently strong, varying from 0.91–1.00, both in the sub scales and for the total ASQ-3 score (see S1 Table).

### Serum samples, thyroid hormones, and iodine status

Serum samples were collected from 288 children and were analyzed for serum thyrotropin (TSH), free thyroxine (FT4), free triiodothyronine (FT3), thyroglobulin (Tg), and antibodies to thyroid peroxidase (TPOAb) and thyroglobulin (TgAb). The samples were analyzed at La Paz University Hospital Foundation for Biomedical Research in Madrid, Spain. The laboratory participates in two quality programs: the internal Unity Interlab control program by BioRad and an external program by the Spanish Society of Laboratory Medicine (SEQCML). A thorough description of the sample collection and analytic procedures has been previously described [20]. Age-dependent reference ranges from the manufacturer were used [20]. The reference ranges were 0.70–6.0 mIU/L for TSH, 12.3–22.8 pmol/L for fT4, and 3.7–8.5 pmol/L for fT3. For the thyroid antibodies TgAb and TPOAb, references of <38 kIU/L and <13 kIU/L were applied, respectively. Tg was considered elevated > 67 μg/L.

Overt and subclinical hypo- and hyperthyroidism have been defined as follows: subclinical hypothyroidism, as TSH above the reference and FT4 within the reference; overt hypothyroidism, as TSH above the reference with FT4 below the reference or highly elevated TSH (>10 mIU/L) and FT4 in the lower quartile; subclinical hyperthyroidism, as TSH below the reference and both FT3 and FT4 within the reference; and overt hyperthyroidism, as TSH below the reference and FT3 or FT4 above the reference ranges. These classifications have also been previously described [20]. The variables “thyroid disturbances”, “TSH outside the references” and “elevated Tg” were used in the statistical analyses. The variable “thyroid disturbance” included hypo- and hyperthyroidism, as well as subjects with normal TSH and FT3 and/or FT4 outside the reference ranges. The variables “TSH outside reference” included children with TSH either below or above the reference range, and “elevated Tg” included children with Tg above the reference range. The three variables described were overlapping; however each was included to explore whether all thyroid disturbances, TSH alone or elevated Tg were predictors of ASQ-3 score. This was decided a priori the statistical analyses. We measured urinary iodine concentration (UIC) from spot samples. Sample collection and analytic procedures have been previously described [20].

### Anthropometrical measures

Body weight of mothers and children were measured to the nearest 100 grams using a UNICEF digital platform scale (SECA 890, Hamburg, Germany). Height was measured to the nearest 0.1 cm using a portable UNICEF length board. Body mass index (BMI) was used to classify body composition among the mothers. The following classifications were used: underweight, normal weight, and overweight/obese, defined by a BMI < 18.5 kg/m2, a BMI = 18.5–24.9 kg/m2, and a BMI ≥ 25.0 kg/m2, respectively [26]. The gender-specific z-scores, height-for-age (HAZ), weight-for-age (WAZ) and weight-for-height (WHZ), were used to classify children’s nutritional status [27], and were calculated using the “WHO macro” for SPSS [28]. The children were categorized as undernourished if HAZ, WAZ, or WHZ was <-2, which is also referred to as stunting, underweight, and wasting, respectively.

### Statistics

Data were analyzed using IBM SPSS version 24 (IBM Corp. Released 2016. IBM SPSS Statistics for Windows, Version 24.0. Armonk, NY: IBM Corp). ASQ-3 scores, total score and subscale scores were categorized at the 33rd percentile, which was decided *a priori*. Logistic regression analyses were used to assess the associations between the ASQ-3 scores, both total scale and subscales, for the respective developmental domains and the different exposure variables: thyroid disturbance, TSH outside reference and elevated Tg. Variables for adjustment were found based on purposeful selection of covariates [29], where the included variables were predetermined. The variables HAZ and WAZ were not included in the model because these variables were considered to be in the causal pathway [20]. The variables that were significantly associated with the dependent variables in the multiple models were used as adjusting variables in the final regression analyses. The following variables were adjusted for in the multiple models: Total ASQ-3 score was adjusted for gender of child and number of toys; Communication subscale, for kindergarten attendance and number of toys; Fine Motor subscale, for gender of child and number of toys; Problem Solving subscale, for education of mother and age of child; and Personal-Social subscale, for age of child and gender of child. None of the pre-defined variables predicted Gross Motor. In post hoc analyses, we discovered a substantial left-skewed distribution in the Gross Motor subscale, indicating a ceiling effect. Furthermore, this domain had poor convergent validity as opposed to the other subscale scores. We decided *a posteriori* to explore the influence of the aforementioned exposure variables on the ASQ-3 total score without the Gross Motor subscale included, hereby called “ASQ-3 score ex. GM”. This was explored by logistic regression models. The ASQ-3 score ex. GM was also included in explorative subgroup analyses. We defined the subgroups as follows: nutritional status indicated by stunting (HAZ<-2), underweight (WAZ<-2), and the thyroid status of mothers measured by thyroid disturbance (yes and no), which was previously published [30]. Thyroid disturbances among the women included both overt- and subclinical hypo- and hyperthyroidism, as well as normal function with positive thyroid antibodies.

### Ethical considerations

Ethical approval for the study was given by the Regional Committees for Medical and Health Research Ethics in Norway (Reference 2013/192), and by the Saharawi Ministry of Public Health. Study information was provided to the participants both orally and in writing. Considering the children’s young age, they were assessed to not have capacity to consent. Written informed consent was given by the mothers on behalf of themselves and their children. Children and women with thyroid disturbances were attended to by local health personnel. The study was conducted according to the principles expressed in the Helsinki Declaration.

## Results

Characteristics of the mothers, children, and home environment are described in Table 1.

**Table 1:** Characteristics of the mothers, children and home environment in a study describing the association between thyroid function and development in young children ^a^.

Characteristics of mothers	*(n = 298)*	Characteristics of children	*(n = 298)*
Age, *years*	33.0 (29.0–38.0)	Age, *months*	31.4 (25.1–35.1)
Height, *cm*	157.3 ± 5.4	Male	142 [47.7]
Weight, *kg*	67.7 ± 13.6	Female	156 [52.3]
BMI, *kg/m*^*2*^	27.3 ±5.2	Breastfed after birth, *yes*	229 [76.8]
< 18.5	9 [3.0]	Still breast fed, *yes*	41 [13.8]
18.5–24.9	94 [31.5]	Weight-for-age, *z-score*	-1.0 ± 0.9
≥ 25	193 [64.8]	<-2 (underweight)	34 [11.4]
Household size, *number*	5.3 ± 1.8	Length/height-for-age, *z-score*	-1.6 ±1.0
Children <5 years	1.5 ± 0.6	<-2 (stunted)	98 [32.9]
Birth complications	63 [21.1]	Weight-for-length/height, *z-score*	-0.2 ± 1.0
Cord around neck	11 [3.7]	<-2 (wasted)	11 [3.7]
C-section	24 [8.1]	Born full term, *yes*	295 [99.0]
Trouble breathing	53 [17.8]	Home environment	*(n = 298)*
Work outside of home	119 [39.9]	Number of toys	
Education level		None	45 [15.1]
None	34 [11.4]	<3	79 [26.5]
Primary school	69 [23.2]	Between 3 and 8	122 [40.9]
Secondary school	129 [43.2]	>8	51 [17.1]
High school	60 [20.1]	Pen and paper available, *yes*	223 [74.8]
Higher education	6 [2.0]	Pen and paper especially for child, *yes*	28 [9.4]
UIC, *μg/L* ^b^	617 (249–1046)	Number of books, *n*	6 (2–10)
		Attend kindergarten, *yes*	53 [17.8]
		Time child plays per week, *hours*	35 (21–42)

^a^ Values are presented as mean ± SD, median (p25-p75), and n [%].

Two missing from anthropometric data of children. One missing from women’s weight and height; 2 missing from women’s BMI; 16 missing from women’s age; 28 missing from total birth complications; One missing from number of toys; 72 missing from pen and paper especially for child; 4 missing number of books.

^b^ Data for UIC were previously published by Aakre et al (30), n = 78.

In Table 2, the prevalence of thyroid dysfunction and iodine status among the children are presented together with the ASQ-scores for the total scale and subscales. In total, 13.9% had a thyroid hormone disturbance, of which 27 children had subclinical hypothyroidism, 1 had subclinical hyperthyroidism, 1 had overt hyperthyroidism, 3 had low FT4, and 8 had elevated FT3 (20). In addition, 13.9% had elevated Tg, in total. None of the children had positive thyroid antibodies. The median iodine concentration in urine was 451.6 μg/L. The median (p25-p75) for total the ASQ-3 score was 180 (150–210), with scores ranging from 45 to 270. For the subscales, the range varied from 0 to 60 in all subscales except for Gross Motor, in which 5 was the minimum score. The different subscale median scores ranged from 28 to 45, where Fine Motor had the lowest median and Gross Motor had the highest. Across all ages, there were 9 constant items, meaning that there were no variations in the scoring for these items, because all the participants had either developed or not developed that specific skill.

**Table 2:** Thyroid disturbances, iodine status and ASQ-3 scores among the children in a study describing the association between thyroid function and child development ^a^.

Thyroid hormones and iodine status	*(n = 288)*
Thyroid disturbances, *%*	40 [13.9]
TSH outside the reference area, *%*	29 [10.1]
Elevated Tg, *%*	40 [13.9]
Iodine status	*(n = 298)*
UIC, *μg/L*	451.6 (273.9–1003.3)
<300, *%*	84 [28.2]
300–599, *%*	97 [32.6]
600–899, *%*	35 [11.7]
≥900 μg/L, *%*	82 [27.5]
ASQ-3 scores	*(n = 298)*
Total score	180 (150–210)
Communication	40 (20–50)
Gross Motor	45 (35–50)
Fine Motor	28 (15–40)
Problem Solving	35 (25–40)
Personal-Social	40 (30–50)

^a^ Values are presented as median (p25-p75) and n [%].

Data regarding thyroid hormones and UIC have been previously presented (20). The scoring range was 0–60 for the subscales and 0–300 for the total score.

### ASQ-scores and thyroid function

Table 3 presents the adjusted coefficients and CIs of covariates detected from crude models, as described in the methods section.

**Table 3:** Associations (adjusted odds ratios (OR) and 95% CI) between covariates and the outcomes in a study describing the association between thyroid function and development in young children.

Possible covariates	Total score	Subscales				
	ASQ-3 score OR (95% CI)	Communication OR (95% CI)	Gross Motor OR (95% CI)	Fine Motor OR (95% CI)	Problem Solving OR (95% CI)	Personal-Social OR (95% CI)
Age mother	-	-	-	-	-	0.97 (0.93, 1.01)
Work status mother	-	-	-	-	1.27 (0.79, 2.04)	-
Work status father	-	-	-	-	-	-
Education mother ^a^	1.88 (0.99, 3.56)	-	-	-	2.07 (0.99, 4.35) ^d^	1.54 (0.82, 2.89)
Education father ^a^	-	-	-	-	-	-
BMI mother	-	-	-	-	-	-
Birth complications ^b^	-	-	-	-	-	-
Breastfeeding status	-	-	-	-	-	-
Breastfed one hour after birth	-	-	-	-	-	-
Household size	-	-	-	-	0.86 (0.75, 1.00)	-
Children under 5	-	-	-	-	-	-
WHZ	-	-	-	-	-	-
Age child	-	-	-	-	0.96 (0.92, 0.99)*	0.96 (0.92, 0.99)*
Gender child	1.85 (1.12, 3.03)*	-	-	1.93 (1.19, 3.15)*	-	2.17 (1.31, 3.61)*
Infections 2 weeks after birth	-	-	-	-	-	-
Diarrhea last two weeks	-	-	-	-	-	-
Other illness last two weeks	-	-	-	-	-	-
Number of toys ^c^	2.21 (1.35, 3.64)*	2.02 (1.21, 3.37)*	-	2.46 (1.51, 4.00)*	1.19 (0.70, 2.02)	1.09 (0.65, 1.82)
Pen and paper available	-	-	-	-	-	-
Pen and paper for child	-	-	-	-	-	-
Attend kindergarten	-	2.60 (1.16, 5.82)*	-	-	1.85 (0.87, 3.95)	-
Number of books	-	-	-	-	-	-
Time child plays per week	-	-	-	-	-	-

Only covariates significantly associated with an outcome in the crude model were included in the multiple regression.

Variables significantly associated with the dependent variable in multiple models are marked with “*”.

Cells marked with “-”, were not significantly associated in crude models and not included in multiple models.

^a^ Categories for education: 0 = None/ primary- or secondary school, 1 = high school or higher education.

^b^ Categories for birth complications: 0 = none, 1 = yes, including cord around neck and trouble breathing.

^c^ Categories for number of toys: 0 = none, 1 = >3

^d^ Education of mother was significantly associated with Problem Solving when re-entered into the model and was therefore used as a covariate in the final model.

The associations between ASQ-3 scores and thyroid disturbances, TSH, and Tg are shown in Table 4. Having a thyroid horomne disturbance or TSH outside the references exhibited a significantly decreased probability of being in the top two tertiles of the ASQ-3 total score in the crude models. In the adjusted models, there was still a negative association, but it was not significant. In the model where the Gross Motor subscale were removed from the total ASQ-3 score, we found a significant and negative association between having a thyroid disturbance and TSH outside the references with being in the top two tertiles of the ASQ-3 score ex. GM, showing adjusted odds ratios (95% CI) of 0.46 (0.23, 0.93) and 0.42 (0.19, 0.94), respectively. For the subscales, Fine Motor was the only variable with a significant association with any of the exposure variables. Children with thyroid disturbances and TSH outside the reference area had a significantly lower probability of being in the highest two tertiles of the ASQ-3 Fine Motor subscale score in the crude models. The association remained significant in the adjusted model for TSH, with an odds ratio (95% CI) of 0.40 (0.18, 0.91). For thyroid disturbances, the association was not significant in the adjusted model (p = 0.067), but the odds ratio had the same direction: 0.52 with a 95% CI of 0.26, 1.10. No associations were observed between any of the ASQ-3 scores and Tg.

**Table 4:** Associations between thyroid disturbances, TSH outside reference and elevated Tg with being in the two upper thirds of the total ASQ-3 score, ASQ-3 score ex. GM and subscales among children.

Dependent variables	Thyroid disturbance				TSH outside reference				Elevated Tg			
	Unadjusted coefficients		Adjusted coefficients		Unadjusted coefficients		Adjusted coefficients		Unadjusted coefficients		Adjusted coefficients	
	OR	95% CI	OR	95% CI	OR	95% CI	OR	95% CI	OR	95% CI	OR	95% CI
Total ASQ-3 score ^a^	0.49	(0.25, 0.97)*	0.51	(0.25, 1.02)^#^	0.40	(0.19, 0.88)*	0.48	(0.22, 1.08) ^#^	0.80	(0.40, 1.58)	0.82	(0.40, 1.67)
ASQ-3 score ex. GM ^a^	0.46	(0.23, 0.89)*	0.46	(0.23, 0.93)*	0.36	(0.16, 0.78)*	0.42	(0.19, 0.94) *	0.83	(0.42, 1.64)	0.85	(0.42, 1.72)
Subscales												
Communication ^b^	0.75	(0.37, 1.51)	0.88	(0.43, 1.83)	0.66	(0.30, 1.45)	0.83	(0.36, 1.88)	0.66	(0.33, 1.32)	0.71	(0.35, 1.44)
Gross Motor ^c^	1.67	(0.76, 3.67)	-		1.48	(0.61, 3.61)	-		1.07	(0.52, 2.22)	-	
Fine Motor ^d^	0.51	(0.26, 0.99)*	0.52	(0.26, 1.10)^#^	0.34	(0.15, 0.74)*	0.40	(0.18, 0.91)*	0.92	(0.46, 1.81)	0.96	(0.47, 1.95)
Problem Solving ^e^	0.79	(0.39, 1.60)	0.86	(0.42, 1.76)	0.69	(0.31, 1.53)	0.76	(0.34, 1.70)	1.03	(0.50, 2.10)	1.17	(0.56, 2.46)
Personal-Social ^f^	0.64	(0.32, 1.25)	0.61	(0.30, 1.21)	0.56	(0.26, 1.21)	0.61	(0.28, 1.35)	0.92	(0.46, 1.83)	0.96	(0.47, 1.94)

*p<0.05

^#^ p = 0.058, p = 0.075, p = 0.067.

Total n = 288. One missing from the variable Number of toys, n = 287 for adjusted coefficients where this variable was included.

^a^ Adjusted for Gender of child (0 = male, 1 = female), Number of toys (0 = < 3, 1 = ≥ 3).

^b^ Adjusted for Kindergarten attendance (0 = no, 1 = yes), Number of toys.

^c^ No adjusting variables

^d^ Adjusted for Gender of child (0 = male, 1 = female), Number of toys.

^e^ Adjusted for Education of mother, Age of child *(months)*.

^f^ Adjusted for Gender of child, Age of child.

### Subgroup analyses

The ASQ-3 score ex. GM was further included in the exploratory subgroup analyses (see S2 Table). Being stunted or underweight did not seem to be associated with a lower ASQ-3 score ex. GM. There was a substantially reduced odds ratio for being in the highest two tertiles of the ASQ-3 score ex. GM, both in the crude and adjusted models for thyroid disturbances and TSH, among children with mothers who had thyroid dysfunction, showing adjusted odds ratios (95% CI) of 0.21 (0.03, 2.10) and 0.08 (0.01, 1.34), respectively. However, the results were not statistically significant, and the group of women with thyroid dysfunction was small, containing only 26 women.

## Discussion

The Lancet Series on child development has estimated that approximately 250 million children under 5 years of age from low- and middle-income countries are at risk of not reaching their developmental potential due to poverty, nutritional inadequacies, and poor learning opportunities [31–33]. Inadequate iodine intake has been listed as one of the main constraints for children in these countries to reach their developmental potential [17]. In our study, we found that children with thyroid disturbances and TSH outside the reference areas were more likely to have lower developmental scores in adjusted regression models, assessed by ASQ-3 score where Gross Motor was excluded. Fine Motor skills was the only domain with a significant association with thyroid hormone status. The median UIC among the children in our sample was above the WHO cut-off of 300 μg/L, which is considered excessive.

The Gross Motor domain was excluded for 3 main reasons. First, we found a ceiling effect in this domain, indicated by a left-skewed distribution, higher median, and low variance. This weakness in the motor domains has also been reported by others [34, 35]. Second, we found low convergent validity of this domain, which is shown in Table 3. Third, the direction of the association in the regression models was opposite from that of the other domains, attenuating the models’ effect estimates.

Others have also discovered associations between thyroid dysfunction and developmental domains. High TSH measured in cord blood was associated with poorer general cognitive and executive functions among healthy boys at 4 years in a Spanish study [36]. In the same study, healthy boys at 10 years of age were included in a follow-up, where associations were found between the highest TSH tertile and lower verbal comprehension and recall skills [37]. A study among Italian infants found a correlation between higher newborn TSH levels and suboptimal neuro-motor outcomes, measured by locomotor skills at 18 months of age [38]. Furthermore, a randomized controlled trial found that iodine supplementation among Albanian children 10–12 years of age improved iodine and thyroid hormone status, as well as cognitive and motor functions [39].

Though the importance of thyroid hormone and iodine sufficiency for child development has been established in the scientific literature, most of the research in this field has focused on in-utero effects of inadequate iodine or thyroid hormone status. Myelination of the brain continues into adult life, and may partially explain the important role of thyroid hormones throughout childhood, as the thyroid hormones are crucial for myelination [40]. In addition, impaired thyroid function can affect metabolism and contribute to the phenomenon of functional isolation [41, 42], where a malnourished child fails to elicit appropriate care and stimulation from the caregiver due to behavior symptoms such as irritability or apathy. Concerning postnatal research, most of the studies are small and have examined newborn TSH either from screening or from cord blood, but not hormonal status in the young child period as explored in this study. Thus, larger studies and more randomized controlled trials are warranted [43].

The Fine Motor subscale was the only domain with a statistically significant association with thyroid disturbances and TSH outside the reference area in our study. This has also been reported by others, such as in the aforementioned Albanian and Italian studies [38, 39]. In a Dutch study, associations were seen between higher levels of thyroid hormones and poorer general motor skills [44]. In contrast, a recent Belgian study found no association between neonatal TSH and psychomotor development of preschool iodine sufficient children [45].

In all the mentioned studies where iodine status was measured, iodine deficiency has been the cause of the thyroid dysfunction. In our study, the children had been living under chronic excessive iodine exposure and a quite high prevalence of thyroid dysfunction was observed [20], with hypothyroidism being the most common. In a review, Leung et al. stated that the underlying mechanisms of iodine-induced hypothyroidism is unclear; however, it may be ascribed to a failure to fully escape from the acute Wolff-Chaikoff effect [6]. Vulnerable groups that may fail to escape from this phenomenon include people with a history of thyroid disease or thyroid autoimmunity and fetuses [6]. How or whether infants and young children may adapt to iodine excess is a subject of little research, and thus the mechanisms are accordingly not well understood. Even though there is evidence that thyroid hormones may be affected similarly by both iodine excess and deficiency [6, 46, 47], to our knowledge, the two studies presented in the introduction are the only studies to have investigated developmental status in association with iodine excess or thyroid disorders caused by iodine excess; moreover, these studies presented contradictory results [18, 19]. Iodine excess may have caused the thyroid dysfunction and hence the lower developmental score in our population, but other causal factors cannot be excluded. Furthermore, it is not possible to draw any causal conclusions based on our cross-sectional study design.

In our study, we found that the associations between poor developmental score and thyroid hormone disturbances were stronger among children of mothers with thyroid dysfunction. As these children’s mothers had a high prevalence of thyroid dysfunction and excessive iodine status [21, 30], the children had probably been exposed to thyroid hormone insufficiencies during the prenatal period. Several studies of women with thyroid hormone insufficiency during pregnancy have found impaired child development and motor skills among their children [48–53]. Motor impairments in adult life have also been seen among children who had congenital hypothyroidism [54, 55]. However, a large and well-conducted randomized trial found no improvement in cognitive function in children 3 years of age after maternal treatment of hypothyroidism at gestation of 15 weeks and 6 days or less [56]. This was confirmed by another recent randomized controlled trial, where treatment for subclinical hypothyroidism or hypothyroxinemia between 8 and 20 weeks gestation did not significantly improve children’s cognitive outcomes at 5 years of age [57]. Both of these trials started treatment relatively late in gestation. A review by Zoeller et al. suggested that thyroid hormone insufficiency at different stages during pregnancy may impair different neurophysiological functions [58]. However, in our study, we cannot be certain of how and when the children were affected by thyroid hormone insufficiency.

### Strengths and limitations of the study

A proportion of the sample population (= 78) was a follow-up population from a previous study, where the sample was selected from convenience sampling [21]. There were no major differences in the demographic characteristics between the initial sample population and the children in the follow-up study. In our sample size calculations, we assumed that approximately 50% would have thyroid dysfunction, based on a previously observed goiter distribution among children [23]. However, the proportion was approximately 14%, which resulted in somewhat lower statistical power. The observed SD was ± 40, which was lower than the anticipated SD of ± 50 from our sample size calculations. The statistical power to detect a difference in total ASQ scores of 20 between the groups was therefore 83%.

The ASQ-3 has not been formally validated in the Saharawi population. However, there were few constant items and a large variability in the scoring both for the total ASQ-3 score and in the different subscales, except for the Gross Motor subscale. This indicated that the screening tool probably captured children with both low scores and high scores, which may suggest that the tool was able to assess developmental differences between the children. Furthermore, we found consistently high ICCs between the examinations throughout the study, which suggests that the examinations were able to assess the same developmental scores. The ASQ has previously been tested in Arabic speaking populations, and has proven to be both internally consistent and culturally sensitive to this population [59]. Other studies have also used translated and culturally adapted ASQ-3 forms, as we did, in similar settings and populations from low- income countries [60–62]. The ASQ-3 has not been validated for use on a continuous scale. Without formally validated cut-offs for poor developmental status, we used the ASQ-3 scores categorized by the 33rd percentile, which has also been done by others [61]. Although our data may be used to examine associations between exposures and developmental status, we cannot determine developmental delays.

It is important to consider that child development may be influenced by several nutritional inadequacies, such as undernutrition, iron, zinc, and B-vitamin deficiencies, as well as deficiencies of essential fatty acids [4], which were not controlled for in this study.

## Conclusions

In this study, thyroid dysfunction was associated with poorer developmental status, as assessed by the ASQ-3 score with the Gross Motor domain excluded. The high iodine intakes may have caused the thyroid hormone disturbances and the delayed developmental status; however, other influential factors cannot be excluded.

## Supporting information

S1 Table Intra class correlations between examiners 1 and 4 for total score and subscale scores during the study. Total number of examiners was 4^a^.
^a^Data are presented as intraclass correlation coefficient and 95% confidence intervals. * = no variation in data, all values had total agreement.
(DOCX)

S2 Table Associations between thyroid disturbances, TSH outside reference and elevated Tg with being in the two upper thirds of the ASQ-3 score ex. GM in different subgroups ^a^.
*p<0.05
^#^ p = 0.051, p = 0.080
One missing from the variable Number of toys, n = 287 for adjusted effects.
^a^ Adjusted for Gender of child (0 = male, 1 = female) and Number of toys (0 = <3, 1 = ≥3).
(DOCX)
