# Supplementary material for: Associations between thyroid dysfunction and developmental status in children with excessive iodine status
Source: PLoS One. 2017 Nov 22;12(11):e0187241. doi: 10.1371/journal.pone.0187241 (PMC5699829; doi:10.1371/journal.pone.0187241)
Supplement: S1 Table — Total number of examiners was 4a. aData are presented as intraclass correlation coefficient and 95% confidence intervals. * = no variation in data, all values had total agreement. (DOCX) [file pone.0187241.s001.docx]

**Supplementary file**

**Supplement 1** Intra class correlations between examiner 1-4 for total score and subscale scores during the study. Total number of examiners was 4^a^

| **Examiners** | **n** | **Total score**  ICC  (95% CI) | **Communication**  ICC  (95% CI) | **Gross motor**  ICC  (95% CI) | **Fine motor**  ICC  (95% CI) | **Problem solving**  ICC  (95% CI) | **Personal social**  ICC  (95% CI) |
| --- | --- | --- | --- | --- | --- | --- | --- |
| Exam. 1and 2 | 8 | 0.97 | 1.00 | 0.97 | 0.98 | 0.99 | 0.95 |
|  |  | (0.87,1.00) | -* | (0.87, 0.99) | (0.90, 1.00) | (0.98, 1.00) | (0.73, 0.99) |
| Exam. 3 and 1 | 6 | 0.99 | 0.95 | 0.94 | 0.98 | 1.00 | 1.00 |
|  |  | (0.94, 1.00) | (0.69, 0.99) | (0.61, 0.99) | (0.87, 1.00) | (0.98, 1.00) | -* |
| Exam. 2 and 3 | 21 | 0.96 | 0.97 | 0.99 | 1.00 | 1.00 | 1.00 |
|  |  | (0.99,1.00) | (0.92, 0.99) | (0.97, 1.00) | (0.96, 1.00) | (1.00, 1.00) | (0.99, 1.00) |
| Exam. 3 and 4 | 6 | 0.99 | 0.96 | 0.95 | 0.97 | 0.91 | 0.98 |
|  |  | (0.95, 1.00) | (0.78, 0.96) | (0.56, 0.99) | (0.82, 1.00) | (0.45, 0.99) | (0.80, 1.00) |
| Exam. 4 and 2 | 11 | 1.00 | 0.99 | 1.00 | 1.00 | 0.97 | 1.00 |
|  |  | (0.99, 1.00) | (0.97, 0.98) | -* | (0.99, 1.00) | (0.89, 0.99) | -* |

^a^Data presented as intra class correlation coefficient and 95% confidence intervals. *= no variation of data, all values had total agreement.
