# Supplementary material for: Associations between thyroid dysfunction and developmental status in children with excessive iodine status
Source: PLoS One. 2017 Nov 22;12(11):e0187241. doi: 10.1371/journal.pone.0187241 (PMC5699829; doi:10.1371/journal.pone.0187241)
Supplement: S2 Table — GM in different subgroups a. *p<0.05 # p = 0.051, p = 0.080 One missing from the variable Number of toys, n = 287 for adjusted effects. a Adjusted for Gender of child (0 = male, 1 = female) and Number of toys (0 = <3, 1 = ≥3). (DOCX) [file pone.0187241.s002.docx]

**Supplement 2 Associations between thyroid disturbances, TSH outside reference and elevated Tg with being in the two upper thirds of the ASQ-3 score ex. GM in different subgroups ^a^**

|  |  | **Thyroid disturbance** | | | | **TSH outside reference** | | | | **Elevated Tg** | | | |
| --- | --- | --- | --- | --- | --- | --- | --- | --- | --- | --- | --- | --- | --- |
|  |  | Unadjusted effects | | Adjusted effects | | Unadjusted effects | | Adjusted effects | | Unadjusted effects | | Adjusted effects | |
| **Subgroups** | n | OR | 95% CI | OR | 95% CI | OR | 95% CI | OR | 95% CI | OR | 95% CI | OR | 95% CI |
| Nutrition status |  |  |  |  |  |  |  |  |  |  |  |  |  |
| Stunted | 95 | 0.60 | (0.21, 1.69) | 0.66 | (0.23, 1.93) | 0.40 | (0.12, 1.31) | 0.42 | (0.12, 1.47) | 0.67 | (0.21, 2.17) | 0.65 | (0.19, 2.22) |
| Not stunted | 191 | 0.46 | (0.18, 1.14) | 0.37 | (0.14, 0.97)* | 0.42 | (0.14, 1.27) | 0.41 | (0.13, 1.29) | 0.90 | (0.38, 2.13) | 0.92 | (0.38, 2.25) |
| Underweight | 33 | 0.50 | (0.08, 2.99) | 0.55 | (0.08, 3.97) | 0.32 | (0.05, 2.24) | 0.41 | (0.05, 3.54) | 0.83 | (0.12, 5.85) | 0.65 | (0.08, 5.44) |
| Not underweight | 254 | 0.45 | (0.22, 0.93)* | 0.45 | (0.21, 0.95)* | 0.36 | (0.16, 0.86)* | 0.42 | (0.17, 1.01) ^#^ | 0.82 | (0.40, 1.71) | 0.87 | (0.41, 1.85) |
| Mother |  |  |  |  |  |  |  |  |  |  |  |  |  |
| Thyroid disturbance | 26 | 0.30 | (0.05, 1.70) | 0.26 | (0.03, 2.10) | 0.09 | (0.01, 0.90)* | 0.09 | (0.01, 1.34) ^#^ | 0.32 | (0.03, 4.09) | 0.48 | (0.25, 9.24) |
| Not thyroid disturbance | 49 | 0.62 | (0.12, 3.12) | 0.59 | (0.11, 3.15) | 0.87 | (1.16, 4.80) | 1.09 | (0.16, 5.28) | 1.21 | (0.24, 6.09) | 1.26 | (0.24, 6.50) |

*p<0.05

^#^ p=0.051, p=0.080

One missing from the variable Number of toys n=287 for adjusted effects.

^a^ Adjusted for gender child (0=male, 1=female) and Number of toys (0=< 3, 1=≥ 3).
